# A Cross‐Sectional Study of Postgraduate Students' Mental Well‐Being: Exploring the Relationship Between Mental Well‐Being, Perceived Stress, Academic Self‐Efficacy, and Self‐Efficacy for Self‐Regulated Learning

**DOI:** 10.1002/hsr2.72429

**Published:** 2026-04-27

**Authors:** Natalie Bisal, Celine Brookes‐Smith, Riya Patel, Sally Sharp, Deborah Lycett, Andy Turner, Maxine Whelan

**Affiliations:** ^1^ Centre for Healthcare and Community Transformation Coventry University Coventry UK; ^2^ Centre for Ethnic Health Research, NIHR Applied Research Collaboration‐East Midlands (ARC‐EM) University of Leicester Leicester UK; ^3^ School of Education, Childhood, Youth and Sport//Faculty of Well‐being, Education and Language Studies The Open University Milton Keynes UK

**Keywords:** academic self‐efficacy, mental well‐being, perceived stress, self‐efficacy for self‐regulated learning

## Abstract

**Background:**

Student well‐being is a growing concern in Higher Education Institutions, yet postgraduate students remain overlooked. Although research has increasingly examined contributing factors to student well‐being, limited attention has been given to the implications of perceived stress and the potential mediating roles of academic self‐efficacy (ASE) and self‐efficacy for self‐regulated learning (SESRL) in postgraduate populations. This study investigated the prevalence of mental health and well‐being outcomes, the association between perceived stress and mental well‐being, and whether ASE and SESRL mediated this relationship among UK postgraduate students.

**Methods:**

UK‐enrolled postgraduate students completed an online survey assessing sociodemographic characteristics, mental health, mental well‐being, perceived stress, ASE, and SESRL. Linear regression examined the association between perceived stress and mental well‐being, and mediation analyses tested whether ASE and SESRL accounted for this relationship.

**Results:**

A total of 115 postgraduate researchers and 219 postgraduate taught students participated. Most students reported moderate levels of perceived stress and mental well‐being, with 11% experiencing high stress and 41% reporting low mental well‐being. Around one‐third met clinical thresholds for moderate‐to‐severe anxiety (32%) or depression (40%). Perceived stress was negatively associated with mental well‐being, and this association was partially mediated by both ASE and SESRL. No differences in stress or mental well‐being were found between postgraduate research and taught students.

**Conclusions:**

Findings are novel in demonstrating that postgraduate students' mental well‐being is shaped by both perceived stress as a primary appraisal and ASE and SESRL as secondary appraisal resources. These results highlight the value of strengthening self‐regulatory efficacy as a means of buffering the negative effects of stress and supporting well‐being across postgraduate taught and research students. Implications for institutional support and resource provision are discussed.

## 1 Introduction

### 1.1 Background

Attending university is well documented as a period of heightened psychological distress [1]. The transition to postgraduate study introduces greater demands for independent study and self‐directed learning exposing students to a wide range of individual, interpersonal, and systemic‐level challenges [2, 3, 4, 5, 6]. These include expectations that distress is a normal part of academic life, experiences of isolation, and pressures related to unsupported and pressured environments [6]. University students commonly report academic performance pressure and elevated levels of stress, anxiety, depression and worry during their studies [7] The COVID‐19 pandemic further intensified these challenges by disrupting access to teaching, supervision, and support, as well as social interaction and academic networking [8]. Collectively, these contextual factors contribute to the broader and well‐recognized concerns surrounding student mental health and well‐being in higher education [9]. A large UK survey found that 40% of more than 21,000 students reported serious personal, emotional, behavioral, or mental health difficulties [7]. Given that almost three million students are enrolled in UK higher education [10], the scale of this problem is substantial. While most research has focused on undergraduate students [6], emerging evidence suggests that postgraduate students also face significant and growing risks to their mental health and well‐being [5].

### 1.2 Mental Well‐Being Among Postgraduate Students

Mental well‐being is a multifaceted phenomenon encompassing multiple interrelated concepts and dimensions, including subjective, psychological, and social components [11]. Two influential perspectives underpin widely used measures such as the Warwick‐Edinburgh Mental Well‐being Scale (WEMWBS [12]). The first is the hedonic perspective, which conceptualizes well‐being in terms of pleasure and happiness [13]. The second is the eudaimonic perspective which emphasizes meaning, self‐realization, and optimal psychological functioning [14]. Although both perspectives remain valuable, recent scholarship argues for moving beyond this dichotomy toward more integrated, multidimensional, and contextually sensitive conceptualizations of well‐being. Such approaches align with transactional perspectives, including the Transactional Model of Stress [15], which emphasize the dynamic interplay between individuals and their environments. From this perspective, well‐being fluctuates depending on the balance between challenges and available resources. It decreases when demands exceed capacity and increases when resources are sufficient [16]. This perspective highlights the inherently dynamic nature of well‐being, shaped by shifting psychological, social, and physical factors in response to life events and situational demands. Evidence indicates that postgraduate students report lower levels of well‐being than the general population [17], with similar findings observed in Belgium [18], France [19] and the United States [20]. Low mental well‐being among university students is associated with poorer academic performance, satisfaction, motivation, and productivity [21, 22, 23, 24].

### 1.3 Stress Among Postgraduate Students

Postgraduate students' stress levels may influence their mental well‐being and academic performance. Stress can be viewed as a dynamic interaction between the individual and their environment [25, 26, 27]. Although stress is considered a normal aspect of student life, reflecting the challenges inherent in learning, development, and growth, individuals differ considerably in the extent to which they experience and evaluate stress. Stress levels may be heightened during difficult periods, as reflected in a survey reporting that 63% of undergraduate and postgraduate students experienced stress at a level that interfered with their daily lives [28]. Similarly, stress has been identified as a major issue affecting the mental health and well‐being of postgraduate researchers [6, 29] who typically face a unique range of academic, financial, personal, and interpersonal challenges [29].

### 1.4 Perceived Stress and the Cognitive Appraisal Theory

Perceived stress has been widely studied in both undergraduate [30] and postgraduate [17] student populations, with consistent evidence demonstrating negative impacts on mental well‐being. Nevertheless, individuals differ in how they evaluate and respond to potential stressors. Some demonstrate adaptive coping, effective stress management, and the ability to maintain positive well‐being despite elevated stress levels [31, 32], whereas others experience poor psychological outcomes [17, 29, 33]. To understand these differences, the present study adopts Cognitive Appraisal Theory [26], which proposes that stress responses arise from two appraisal processes. Primary appraisal involves evaluating whether a situation is threatening or stressful, or alternatively challenging but manageable; this process corresponds to perceived stress. During secondary appraisal, individuals evaluate whether they possess the skills and resources necessary to cope with the situation [26]. Together, these appraisals influence emotional and behavioral outcomes, as individuals' perception of the stressful situation depends both on what they interpret as stressful and on their perceived capacity to respond appropriately [25]. Coping thus functions as a regulatory mechanism that helps rebalance perceived demands and perceived resources [25].

### 1.5 Self‐Efficacy in Academic Contexts

Within academic environments, self‐efficacy plays a central role in secondary appraisal processes. In line with Bandura's Social Cognitive Theory [34], self‐efficacy reflects students' beliefs in their ability to utilize cognitive, behavioral, and emotional resources and coping skills to manage academic challenges [35]. These beliefs align closely with secondary appraisal by shaping perceptions of controllability and coping capacity. Research shows that self‐efficacy plays a key role in student behavior [36] and functions as an important psychological resource that underpins individuals' perceived ability to influence events and exert control over their environment [37, 38]; a construct that closely aligns with appraisal processes in Cognitive Appraisal Theory [34]. Low self‐efficacy has been linked to burnout [22], greater perceived stress [39, 40, 41], and psychological distress [42] in higher education students. Furthermore, several studies have demonstrated support for the mediating role of self‐efficacy on psychological outcomes, such as life satisfaction and perceived stress, in undergraduate students [43, 44, 45].

### 1.6 Academic Self‐Efficacy and Self‐Efficacy for Self‐Regulated Learning

Both academic self‐efficacy (ASE) and self‐efficacy for self‐regulated learning (SESRL) are theoretically and contextually important academic self‐beliefs. ASE refers to students' confidence in their ability to meet academic demands [46] and has been positively associated with well‐being [47] and negatively associated with perceived stress [39, 41] in university students. ASE is also consistently linked to greater motivation, engagement, persistence, academic performance [48], and use of cognitive and self‐regulatory strategies [49, 50]. Students with higher ASE are more likely to anticipate that their efforts will lead to successful outcomes [51], reflecting stronger expectancy beliefs and more positive academic appraisals. Such expectancy beliefs may indirectly reduce the intensity of primary appraisals of academic demands by enhancing students' perceived control and coping capacity [52], thereby strengthening secondary appraisal processes. Consequently, ASE may play an important role in shaping perceived control and coping flexibility in relation to academic stress [36, 51, 53, 54], contributing to improved well‐being. Students with greater confidence tend to report greater happiness [55].

Self‐efficacy for self‐regulated learning refers to students' beliefs in their capability to effectively implement self‐regulatory learning strategies [56]. SESRL represents a second theoretically grounded secondary appraisal mechanism, as it influences perceived coping capacity through students' confidence in their ability to manage the learning process. SESRL supports students in adopting key self‐regulatory behaviors, such as planning, monitoring and adjusting learning strategies [57], and has been shown to positively influence academic motivation, persistence, and achievement [58, 59, 60]. Research further demonstrates that higher self‐regulatory efficacy supports more adaptive cognitive and affective responses, such as the use of positive reappraisal [51], and facilitates greater autonomy, self‐direction, strategy adjustment, and adaptive coping [51, 60, 61, 62]. SESRL is particularly relevant for postgraduate students, who must navigate high levels of independent and self‐directed study. Despite this relevance, no empirical research has examined the role of SESRL in the relationship between perceived stress and mental well‐being in postgraduate students. Given the prevalence of concerns, such as academic performance [63], lack of confidence in academic abilities [64], imposter feelings [17, 18] and disappointment in one's capabilities [64, 65], SESRL represents a salient mechanism shaping how postgraduate students appraise and cope with academic stress.

**Figure 1:**
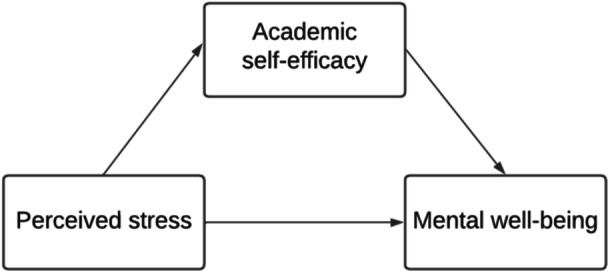
Conceptual model on the mediating role of postgraduate students’ ASE in the relationship between perceived stress and mental well‐being. ASE, academic self‐efficacy.

**Figure 2:**
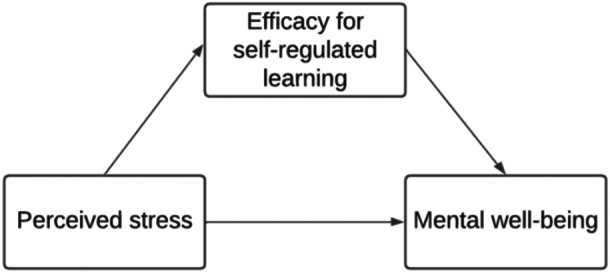
Conceptual model on the mediating role of postgraduate students' SESRL in the relationship between perceived stress and mental well‐being. SESRL, self‐efficacy for self‐regulated learning.

Together, these pathways suggest a transactional process whereby perceived stress influences mental well‐being through two distinct but complementary forms of secondary appraisal: ASE, which shapes students' perceived capability to meet academic demands, and SESRL, which shapes students' capacity to regulate the learning process, thus reflecting different aspects of postgraduate students' perceived ability to regulate and manage their own learning. Higher efficacy in both domains increases the likelihood of engaging in adaptive coping behaviors (e.g., planning, effort regulation), which may reduce perceived discrepancies between academic demands and coping resources. In doing so, ASE and SESRL may reduce the overall impact of perceived stress on mental well‐being. Based on Lazarus and Folkman's cognitive appraisal model, this study therefore examined how perceived stress (*primary appraisal*) influenced academic self‐efficacy (Figure 1) and self‐efficacy for self‐regulated learning (Figure 2) (*secondary appraisal*), and in turn affected mental well‐being among postgraduate taught and research students.

## 2 Materials and Methods

This study was written in accordance with STROBE guidelines [66].

### 2.1 Study Design and Setting

A cross‐sectional survey design exploring university student mental health and well‐being, perceived stress, ASE, and SESRL. The survey was open via Qualtrics from February to July 2021. Participants were able to enter themselves into a prize draw offering shopping vouchers to incentivise participation.

### 2.2 Ethics Statement

Ethics approvals were obtained from Coventry University's Research Ethics Committee (ref P116128). Participants were provided with study information before consent was obtained.

### 2.3 Eligibility and Recruitment of Participants

Participants were eligible if aged 18 years or older, a current student enrolled at Coventry University in academic year 2021, and able to provide informed consent to participate. Both undergraduate and postgraduate students were able to take part, with the present study focusing only on postgraduate students (both taught [i.e., MSc students] and research [i.e., PhD or doctoral students]). Participants were invited to take part via advertisements (study materials and a direct survey link) shared digitally at the university through newsletters and internal communications.

### 2.4 Data Collection Procedure

After clicking on the direct survey link and completing the consent form, participants were directed to the survey comprising 49 questions (approximately 25 min to complete) which related to the following areas.

#### 2.4.1 Sociodemographic, Academic and Mental Health Characteristics

Sociodemographic measures included age, gender, ethnic background, sexual orientation, religion, faith or belief, marital status, employment status (alongside studies), parental education level (as a measure of socioeconomic status), caring responsibilities, and disability, illness or health condition. Academic‐related measures included study level (undergraduate, postgraduate taught, postgraduate research), fee status (UK‐home, international within EU, or international outside EU), academic year, mode of study (part‐time or full‐time), and faculty or subject area.

#### 2.4.2 Depression

The Patient Health Questionnaire [67] (PHQ‐9) was used as a screening measure for depression. The scale contains nine items rated on a 4‐point Likert scale from 0 (not at all) to 3 (nearly every day). Higher scores on the PHQ‐9 indicated greater levels of depression. PHQ‐9 scores were categorized into none to minimal (0–4), mild (5–9), moderate (10–14), moderately severe (15–19), and severe (20–27) depressive symptoms. This scale is widely used in UK primary healthcare as a screening tool for depression [67] and has good internal consistency (*α* = 0.89). Cronbach's alpha for the current study was 0.86.

#### 2.4.3 Anxiety

The General Anxiety Disorder scale [68] (GAD‐7) was used as a screening measure for anxiety. The scale contains seven items rated on a 4‐point Likert scale from 0 (not at all) to 3 (nearly every day). Higher scores on the GAD‐7 indicated greater levels of anxiety. GAD scores were categorized into minimal (0–4), mild (5–9), moderate (10–14), and severe (15–21) anxiety symptoms. As with the PHQ‐9, this scale is widely used in UK primary healthcare as a screening tool for anxiety/GAD [68] and has good internal consistency (α = 0.92). Cronbach's alpha for the current study was 0.91.

#### 2.4.4 Mental Well‐Being

The Warwick‐Edinburgh Mental Well‐being Scale [12] (WEMWBS) was used to measure hedonic and eudemonic aspects of mental well‐being, focusing on experiences during the preceding 2 weeks. The scale contains 14 items rated on a 5‐ point Likert scale from 1 (none of the time) to 5 (all of the time), with higher scores indicating better mental well‐being. Total possible scores ranged from 14 to 70 and categorized into low (14‐42), moderate (43‐59) and high (60–70) well‐being. The WEMWBS scale has been widely used and validated in university students (39, 60–64) with good internal consistency (α = 0.89–0.92 [12, 69]). Cronbach's alpha for the current study was 0.92.

#### 2.4.5 Stress

The Perceived Stress Scale [70] was used to measure perceived stress, asking about feelings and thoughts in relation to stressful situations in the preceding month. The scale contains ten items rated on a 5‐point Likert scale from 0 (never) to 4 (very often), with higher scores indicating greater perceived stress. PSS‐10 scores were categorized into low (0–13), moderate (14–26) and high (27–40) perceived stress. PSS‐10 is a reliable tool to measure different aspects of perceived stress showing good internal consistency (α = 0.74–0.91) in university students and adult populations [71]. Cronbach's alpha in this study was 0.81.

#### 2.4.6 Academic Self‐Efficacy

The ASE scale was adapted from Chemers et al [72] and used to measure confidence in ability to perform well academically. The scale contains eight items rated on a 7‐point Likert scale from 1 (very untrue) to 7 (very true). Higher scores indicated greater ASE. For the present study, three items were adapted to better suit the university academic student context: “I know how to study to perform well on tests” was changed to “…on exams/assignments,” “I usually do very well in school and at academic tasks” was changed to “…in university and at academic tasks” and “I am very capable of succeeding at this college” was changed to “…at this university”. The scale was found to have good internal consistency (α = 0.81 [72];). Cronbach's alpha for the current study was 0.91.

#### 2.4.7 Self‐Efficacy for Self‐Regulated Learning

The SESRL scale was adapted [73] to measure perceived self‐efficacy for implementing a variety of self‐regulated learning skills and strategies. The scale contains eleven items rated on a 5‐point Likert scale from 1 (no confidence at all) to 5 (complete confidence). Higher scores indicated greater perceived efficacy for self‐regulated learning. For the present study, seven items were adapted to better suit the university academic student context: “Finish homework assignments by deadlines?” was changed to “Finish assignments…,” “Concentrate on school subjects” became “…on course subjects”, “Use the library to get information for class assignments?” became “…for assignments?,” “Plan your schoolwork?” became “…your university work?,” “Organize your schoolwork” became “…your university work,” “Remember information presented in class and textbooks” became “…in teaching and textbooks,” and “Motivate yourself to do schoolwork” became “…to do university work.” The scale was found to have good internal consistency (α = 0.87 [73];). Cronbach's alpha for the current study was 0.92.

#### 2.4.8 Impact of COVID‐19 on Well‐Being

Participants rated their subjective mental health and well‐being in relation to the coronavirus pandemic by the question: “How would you describe your mental health and well‐being currently, compared to before the coronavirus pandemic?” assessed on a 5‐point Likert scale from 0 (‘very much worse’) to 4 (‘very much better)’.

### 2.5 Data Analysis

Data were analysed using SPSS version 26.0. Participant responses were included if 96% of items were complete. Descriptive statistics (frequencies, percentages, mean, standard deviation) were calculated. Independent‐samples *t*‐tests assessed for differences in scores between postgraduate taught and postgraduate researchers. The statistical assumptions for linear regression and mediation were found to be acceptable after data were assessed for outliers (using boxplots and casewise diagnostic statistics using a cut‐off of 3 standard deviations), normality of residuals (using normal probability plots, histograms, and scatter plots), linearity and homoscedasticity (using visual inspection of graphs plotting standardized residuals against predicted values), and independence of residuals (accepted with Durbin‐Watson statistic of 1.52). Preliminary analyses examined associations between sociodemographic or academic characteristics and mental well‐being; significantly associated variables were included as covariates in subsequent models.

Scatterplots assessed the relationships between mental well‐being and perceived stress, ASE, and SESRL. Pearson's bivariate correlations were calculated. Effect sizes of correlation coefficients followed Cohen's definitions of small (*r* = 0.10), medium (*r* = 0.30), and large (*r* = 0.50) [74, 75]. Linear regressions explored the association between perceived stress and mental well‐being, with models entered in theory‐driven blocks and presented using stepwise methods to illustrate incremental contribution of each construct. This approach reflects the conceptual framework in which ASE and SESRL are positioned as secondary appraisal resources that follow perceived stress and influence mental well‐being. Standardized regression coefficients (*β*) were interpreted using Cohen's conventions for correlation‐based effect‐sizes: small (0.10), medium (0.30), large (0.50) [76]. We used the common heuristic of 15 participants per independent variable as an initial indication that the sample size was adequate for regression models.

Mediation analyses assessed two models to determine whether ASE (Model 1) or SESRL (Model 2) mediated the relationship between perceived stress and mental well‐being. Models were adjusted for covariates identified in preliminary analyses. To test mediation, four regression models were estimated: (1) effect of the independent variable on the outcome (path c), (2) effect of the independent variable on the mediator (path a), (3) effect of the mediator on the outcome (path b), and 4) indirect effect of the independent variable on the outcome when controlling for the mediator variable [77, 78, 79, 80]. Mediation was deemed present if zero did not fall within the bias‐corrected, accelerated 95% confidence interval, BCa 95% CI [78, 81]. Analyses were conducted using the PROCESS macro for SPSS (version 26.0) developed by Hayes [82] to calculate the mediation models with 5000 bootstrap samples [79]. Percent mediation (PM) was calculated to estimate the proportion of the total effect accounted for by each mediator [83]. Two‐tailed *p* < 0.05 were considered statistically significant.

### 2.6 Patient and Public Involvement

Six university students (three females, three males; aged between 22 and 47 years) helped to pilot the survey and input on the design and phrasing. The public contributors checked the survey in terms of measures and understanding of instructions, determined time taken and reflected on acceptability of the time taken to complete the survey. There was a clear need to reword and clarify items on the scale for SESRL.

## 3 Results

### 3.1 Participant Characteristics

Four hundred and thirty responses were recorded, and 95 responses were removed due to being incomplete, duplicated or having presented doubtful data. The final sample comprised 334 participants (taught: *n* = 219, 65.6%; research: *n* = 115 34.4%). Participants were on average 30.3 (SD = 9.0) years old, ranging from 20 to 80 years. Half of participants were ‘international outside the EU’ (*n* = 171, 51.2%), while a larger proportion were in their first year (*n* = 230, 68.9%) and studied full‐time (*n* = 261, 78.1%). Most participants reported studying in the subject area of health and life sciences (*n* = 153, 45.9%) (Table 1). More than half of participants identified as female (53.6%) and most reported being heterosexual (85.6%). A large proportion of participants were from Asian (39.8%) or white (38%) ethnic backgrounds.

**Table 1:** Sociodemographic and academic characteristics of participants.

Variable	Whole sample (*n* = 334)	Postgraduate taught (*n* = 219)	Postgraduate research (*n* = 115)
Age, mean (SD)	30.3 (9.0)	29.2 (8.1)	32 (10.2)
Gender, *n* (%)			
Male	148 (44.3%)	108 (49.3%)	40 (34.8%)
Female	179 (53.6%)	108 (49.3%)	71 (61.7%)
Non‐binary	1 (0.3%)	1 (0.5%)	0
Intersex	2 (0.6%)	1 (0.5%)	1 (0.9%)
Transgender	1 (0.3%)	0	1 (0.9%)
Prefer not to say	3 (0.9%)	1 (0.5%)	2 (1.8%)
Ethnic background, *n* (%)			
White or white other	127 (38.0%)	67 (30.6%)	60 (52.5%)
Asian or Asian British	133 (39.8%)	103 (47.0%)	30 (26.1%)
Black, African, Caribbean or Black British	38 (11.3%)	29 (13.2%)	9 (7.8%)
Mixed or multiple	10 (3.0%)	6 (2.7%)	4 (3.5%)
Other (incl. Arab)	21 (6.3%)	13 (5.9%)	8 (7.0%)
Prefer not to say	5 (1.5%)	1 (0.5%)	4 (3.5%)
Sexual orientation, *n* (%)			
Heterosexual	286 (85.6%)	195 (89.0%)	91 (79.1%)
Gay, lesbian or bisexual	34 (10.2%)	17 (7.8%)	17 (14.8%)
Other	3 (0.9%)	1 (0.5%)	2 (1.7%)
Prefer not to say	11 (3.3%)	6 (2.7%)	5 (4.3%)
Religion, faith or belief, *n* (%)			
Atheist	54 (16.2%)	26 (11.9%)	28 (24.3%)
Christian^a^	86 (25.7%)	58 (26.5%)	28 (24.3%)
Muslim	55 (16.5%)	40 (18.3%)	15 (13.0%)
Hindu	50 (15.0%)	43 (19.6%)	7 (6.1%)
Spiritual but not religious	34 (10.2%)	18 (8.2%)	16 (13.9%)
Agnostic	23 (6.9%)	16 (7.3%)	7 (6.1%)
Sikh	7 (2.1%)	5 (2.3%)	2 (1.7%)
Buddhist	4 (1.2%)	3 (1.4%)	1 (0.9%)
Jewish	2 (0.6%)	1 (0.5%)	1 (0.9%)
Any other religion	3 (0.9%)	1 (0.5%)	2 (1.7%)
Prefer not to say	16 (4.8%)	8 (3.7%)	8 (7.0%)
Marital status^b^, *n* (%)			
Single	146 (44.1%)	111 (50.7%)	35 (30.4%)
In a relationship or partnered	90 (27.2%)	46 (21.0%)	44 (38.3%)
Married or in a civil partnership	87 (26.3%)	58 (26.5%)	29 (25.2%)
Other	3 (0.9%)	3 (1.4%)	0
Prefer not to say	5 (1.5%)	1 (0.5%)	4 (3.5%)
Employment status^b^, *n* (%)			
None	134 (40.5%)	88 (40.2%)	46 (40.7%)
Full‐time	59 (17.8%)	35 (16.0%)	24 (21.2%)
Part‐time	93 (28.1%)	73 (33.3%)	20 (17.7%)
Zero hours contract	17 (5.1%)	13 (5.8%)	4 (3.5%)
Casual basis	16 (4.8%)	4 (3.7%)	12 (10.6%)
Other	12 (3.6%)	5 (2.3%)	7 (6.2%)
Parental education level, *n* (%)			
No formal qualifications	38 (11.4%)	22 (10.0%)	16 (13.9%)
Qualifications below degree level	83 (24.9%)	49 (22.4%)	34 (29.6%)
At least one parent had a degree level qualification	196 (58.7%)	134 (61.2%)	62 (53.9%)
Don't know (or other)	7 (2.1%)	6 (2.7%)	1 (0.9%)
Prefer not to say	10 (3.0%)	8 (3.7%)	2 (1.7%)
Fee status, *n* (%)			
UK citizen	129 (38.6%)	69 (31.5%)	60 (52.5%)
EU student	34 (10.2%)	21 (9.6%)	13 (11.3%)
International student (outside EU)	171 (51.2%)	129 (58.9%)	42 (36.5%)
Academic year, *n* (%)			
First	230 (68.9%)	169 (77.2%)	61 (53.0%)
Second	35 (10.5%)	22 (10.0%)	13 (11.3%)
Third	22 (6.6%)	7 (3.2%)	15 (13.0%)
Fourth	24 (7.2%)	7 (3.2%)	17 (14.8%)
Fifth or above	23 (6.9%)	14 (6.4%)	9 (7.8%)
Faculty or subject area^c^, *n* (%)			
Health and life sciences	153 (45.9%)	106 (48.4%)	47 (40.9%)
Engineering, environment, and computing	130 (39.0%)	94 (42.9%)	36 (31.3%)
Business and law	17 (5.1%)	9 (4.1%)	8 (7.0%)
Arts and humanities	20 (6.0%)	9 (4.1%)	11 (9.6%)
Other	13 (3.9%)	1 (0.5%)	12 (10.4%)

Abbreviations: EU, European Union; PhD, Doctor of Philosophy; UK, United Kingdom.

^a^ Included Church of England, Catholic, Protestant and all other Christian denominations.

^b^ Total responses for this item were *n* = 331.

^c^ Total responses for this item were *n* = 333.

### 3.2 Rates of Mental Health, Mental Well‐Being, Perceived Stress, ASE and SESRL

Four in ten (*n* = 136, 40.7%) participants reported low mental well‐being, whilst 52.7% (*n* = 176) had moderate mental well‐being (Table 2). Thirty‐five (10.5%) participants reported experiencing high perceived stress, with most (*n* = 232, 69.5%) reporting moderate perceived stress. Three in ten (31.6%) participants were classified as experiencing moderate‐to‐severe anxiety symptoms, reaching the clinical cut‐off thresholds. More than one third (39.7%) of participants reached the clinical cut‐off point threshold for depression. All measures were deemed to have good internal reliability.

**Table 2:** Descriptive statistics for mental health and well‐being outcomes for all participants and separated by postgraduate researchers and postgraduate taught students.

Variable	Whole sample (*n* = 334)	Postgraduate taught (*n* = 219)	Postgraduate researchers (*n* = 115)
Mental well‐being^a^, M(SD)	44.96 (9.83)	45.59 (9.83)	43.75 (9.75)
Perceived stress^b^, M(SD)	19.01 (6.43)	18.75 (6.55)	19.51 (6.20)
Anxiety^c^, M(SD)	7.31 (5.54)	7.13 (5.79)	7.64 (5.08)
Depression^d^, M(SD)	8.80 (5.75)	8.81 (5.96)	8.77 (5.40)
Academic self‐efficacy^e^, M(SD)	41.49 (9.11)	39.94 (9.54)	44.44 (7.43)
Self‐efficacy for self‐regulated learning^f^, M(SD)	36.92 (8.75)	36.25 (9.25)	38.19 (7.58)
*Cut‐offs*			
WEMWBS, *n* (%)			
*Low*	136 (40.7%)	79 (36.1%)	57 (49.6%)
*Moderate*	176 (52.7%)	123 (56.3%)	53 (46.1%)
*High*	22 (6.6%)	17 (8.0%)	5 (4.5%)
PSS‐10, *n* (%)			
*High*	35 (10.5%)	25 (11.6%)	10 (8.7%)
*Moderate*	232 (69.5%)	149 (67.8%)	83 (72.2%)
*Low*	67 (20.0%)	45 (20.7%)	22 (19.1%)
GAD‐7, *n* (%)			
*Minimal*	128 (38.0%)	92 (41.9%)	34 (29.6%)
*Mild*	102 (30.3%)	57 (26.1%)	45 (39.2%)
*Moderate*	63 (18.7%)	40 (18.3%)	22 (19.0%)
*Severe*	44 (13.1%)	30 (13.7%)	14 (12.1%)
PHQ‐9, *n* (%)			
*None*	20 (5.9%)	13 (5.9%)	5 (4.3%)
*Minimal*	71 (21.2%)	50 (22.8%)	21 (18.3%)
*Mild*	112 (33.2%)	67 (30.6%)	45 (39.2%)
*Moderate*	78 (23.1%)	50 (22.8%)	28 (24.3%)
*Moderately severe*	41 (12.2%)	29 (13.3%)	11 (9.6%)
*Severe*	15 (4.5%)	10 (4.8%)	5 (4.4%)

Abbreviation: SD, standard deviation.

^a^ Using the WEMWBS; higher scores meaning higher mental well‐being, possible range 14–70.

^b^ Using the PSS‐10; higher scores meaning higher perceived stress, possible 0–40.

^c^ Using the GAD‐7; higher scores meaning greater anxiety, possible range 0–21.

^d^ Using the PHQ‐9; higher scores meaning greater depression, possible range 0–27.

^e^ Using the academic self‐efficacy scale; higher scores meaning higher academic self‐efficacy, possible range 8–56.

^f^ Using the self‐efficacy for self‐regulated learning scale; higher scores meaning higher self‐efficacy for self‐regulated learning, possible range 11–55.

### 3.3 Differences Between Postgraduate Taught and Research

No significant differences were identified between postgraduate taught and postgraduate research students in mental well‐being (*t*(332) = 1.63, *p* = 0.10) or perceived stress (*t*(332) = 1.03, *p* = 0.31). Postgraduate students were subsequently combined in the analyses.

### 3.4 Relationships between Mental Well‐Being, Perceived Stress, ASE and SESRL

Pearson's correlations showed that mental well‐being was strongly negatively correlated with perceived stress (large effect) and positively correlated with ASE (medium effect) and SESRL (large effect) (Table 3). Perceived stress negatively correlated with ASE (medium effect) and SESRL (large effect).

**Table 3:** Means, standard deviations, and Pearson's correlations among mental well‐being, perceived stress, academic self‐efficacy and self‐efficacy for self‐regulated learning.

Variable	M	SD	1	2	3
Mental well‐being	44.96	9.83	—		
Perceived stress	19.01	6.43	−0.669**	—	
Academic self‐efficacy	41.49	9.11	0.380**	−0.363**	—
Self‐efficacy for self‐regulated learning	36.92	8.75	0.585**	−0.520**	0.682**

*Note:* Correlations are Pearson's *r*.

Abbreviations: *M* = mean, SD = standard deviation.

** *p* < 0.01 (two‐tailed).

### 3.5 Covariate Analyses

Gender, ethnic background, sexual orientation, marital status, ‘health condition, illness or disability’, and fee status were associated with mental well‐being.

### 3.6 Scatterplots

**Figure 3:**
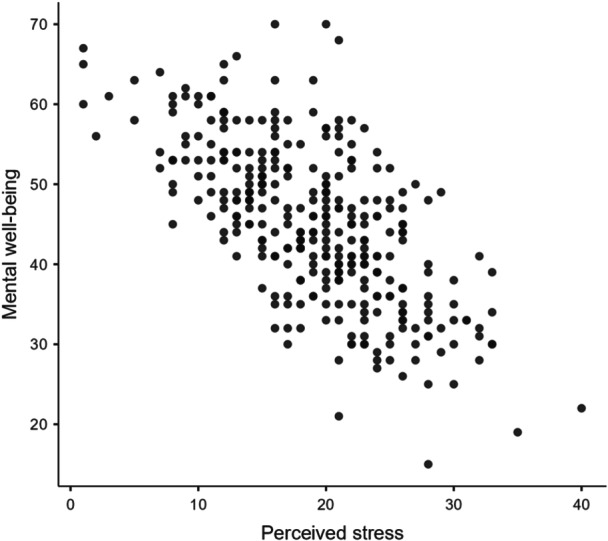
Scatterplot graph for mental well‐being and perceived stress.

**Figure 4:**
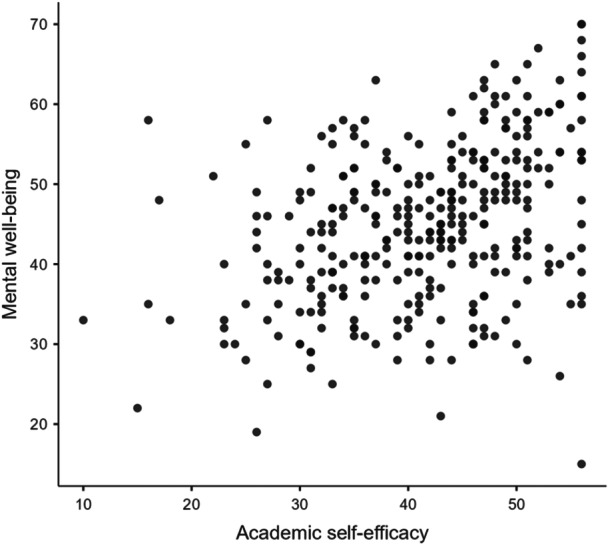
Scatterplot graph for mental well‐being and academic self‐efficacy.

**Figure 5:**
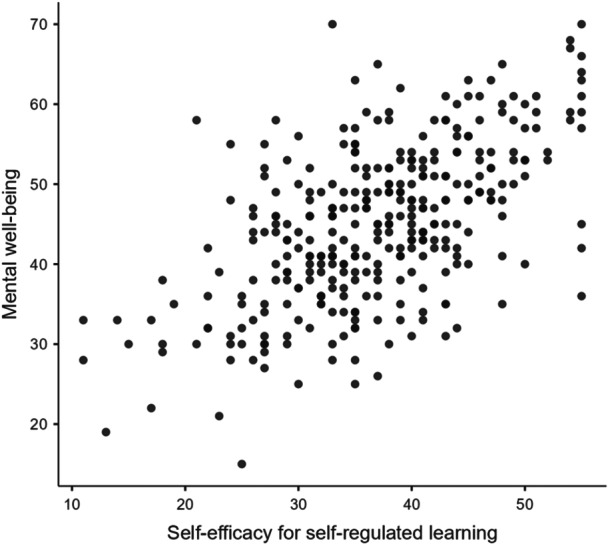
Scatterplot graph for mental well‐being and self‐efficacy for self‐regulated learning.

Scatterplot diagrams showed relatively linear relationships between mental well‐being and the main variables: perceived stress (Figure 3), ASE (Figure 4) and SESRL (Figure 5).

### 3.7 Association Between Perceived Stress and Mental Well‐Being

Perceived stress was negatively associated with mental well‐being (*B* = −1.02, 95% CI [−1.15, −0.90], *p* < 0.001). After adjustment, perceived stress remained negatively associated (*B* = −1.01, 95% CI [−1.13, −0.88], *p* < 0.001). Academic self‐efficacy was added and this positively associated with mental well‐being. The effect of perceived stress on mental well‐being reduced (*B* = −0.89, 95% CI [−1.02, −0.76], *p* < 0.001). SESRL was added and positively associated with mental well‐being. The effect of perceived stress on mental well‐being reduced (*B* = −0.75, 95% CI [−0.89, −0.62], *p* < 0.001). Participants decreased by 0.75 points in mental well‐being for each additional point in perceived stress (medium effect; *β* = −0.49).

### 3.8 Factors Mediating the Relationship Between Perceived Stress and Mental Well‐Being

**Figure 6:**
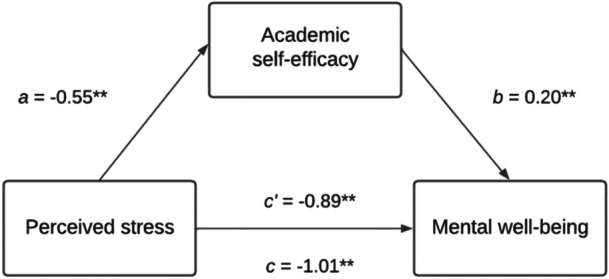
Mediation analysis for academic self‐efficacy on the association between perceived stress and mental well‐being. *N* = 334; c' refers to the direct effect; c refers to the total effect; **Pathway is significant at the 0.001 level; Mediation model adjusted for gender, ethnic background, sexual orientation, marital status, disability, health condition, or illness, fee status. The relationship between perceived stress and mental well‐being was partially mediated by SESRL with a significant indirect effect of perceived stress on mental well‐being through SESRL (*ab* = −0.26, BCa 95% CI [−0.36, −0.18]). In the final adjusted mediation model (Figure 7), an indirect association was found (*ab* = −0.26, BCa 95% CI [−0.35, −0.17]) and SESRL accounted for 25% of the total effect, *P*_M_ = 0.25.

The relationship between perceived stress and mental well‐being was partially mediated by ASE illustrated by a significant indirect effect of perceived stress on mental well‐being through ASE (*ab* = −0.09, BCa 95% CI [−0.16, −0.03]). In the final adjusted mediation model (Figure 6), an indirect association was found (*ab* = −0.11, BCa 95% CI [−0.18, −0.05]) with ASE accounting for 11% of the total effect, *P*_M_ = 0.11.

**Figure 7:**
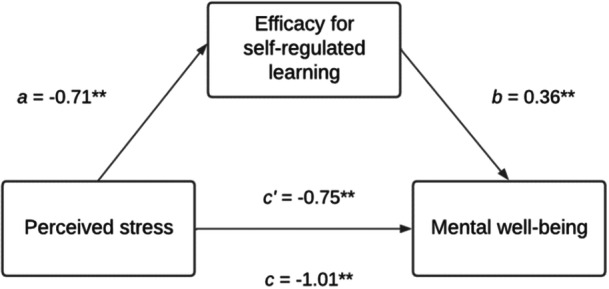
Mediation analysis for self‐efficacy for self‐regulated learning on the association between perceived stress and mental well‐being. *N* = 334; c' refers to the direct effect; c refers to the total effect; **Pathway is significant at the 0.001 level; Mediation model adjusted for gender, ethnic background, sexual orientation, marital status, disability, health condition, or illness, fee status.

### 3.9 COVID‐19 Pandemic Impact on Well‐Being

Participants reported mental health and well‐being compared to before the pandemic as ‘very much worse or somewhat worse’ (*n* = 155, 46.6%), ‘about the same’ (*n* = 111, 33.2%), ‘somewhat better or very much better’ (*n* = 68, 20.4%).

## 4 Discussion

### 4.1 Summary

This cross‐sectional study examined mental well‐being, perceived stress, and the mediating roles of academic self‐efficacy (ASE) and self‐efficacy for self‐regulated learning (SESRL) among over 300 UK postgraduate students. Consistent with the Cognitive Appraisal Theory [26] and Social Cognitive Theory [34], higher perceived stress was associated with poorer mental well‐being and both ASE and SESRL partially mediated this relationship. No differences were observed between postgraduate taught and research students in perceived stress or mental well‐being.

### 4.2 Mental Well‐Being and Perceived Stress

Mental well‐being in this sample was lower than levels reported in the wider university population [84] and general population [12, 69], aligning with averages observed among postgraduate researchers [85]. Rates of anxiety, depression, and moderate‐to‐high stress were consistent with recent UK postgraduate data [6], and likely influenced by the COVID‐19 pandemic period [86]. Our findings reinforce previous research showing that perceived stress is strongly and negatively associated with mental well‐being [45, 53, 87, 88]. According to Cognitive Appraisal Theory [26], stress occurs when individuals perceive environmental demands as exceeding their coping resources. Postgraduate students must navigate numerous academic and personal stressors [89], and when appraised as threatening or uncontrollable, well‐being is negatively impacted [26]. Prior studies have also linked higher perceived stress to maladaptive coping [90], contributing further to negative effects of stress on mental well‐being. These findings underscore the need for interventions that reduce perceived stress and/or enhance students’ coping resources [6], two pathways central to the theoretical framework underpinning this study.

Contrary to previous research [6, 18, 91], no differences were observed between taught and research students. Pandemic‐related disruptions, such as remote learning, reduced peer interaction, altered academic expectations, and shifts to online delivery, may have contributed to comparable experiences of isolation and uncertainty across both groups [92, 93]. As a result, typical distinctions in the nature and appraisal of stressors between taught and research students may have been attenuated [8, 93, 94, 95].

### 4.3 Mediating Roles of ASE and SESRL

The associations between perceived stress, ASE, SESRL, and mental well‐being suggest that postgraduate psychological functioning is shaped by both the demands of postgraduate study and students' internal appraisals of their academic capabilities. In line with Cognitive Appraisal Theory [26], ASE and SESRL reflect secondary appraisal processes through which students evaluate their perceived capacity to manage academic and personal challenges. Students who believe they can meet academic demands (ASE) and regulate their learning effectively (SESRL) are more likely to view stressors as manageable, reducing distress and supporting well‐being [26]. These findings extend evidence from undergraduate populations [47, 72], by demonstrating that postgraduate well‐being is influenced not only by the volume of demands students face but also by their perceived ability to cope with those demands. The observed mediating roles of ASE and SESRL align with work linking perceived stress to ASE [39, 41], ASE to psychological outcomes [47], and general self‐efficacy to well‐being outcomes [45]. Together, these patterns highlight the importance of academic‐related efficacy beliefs in shaping how students interpret stressors and the degree to which stress impacts well‐being.

Although both ASE and SESRL were significant mediators, SESRL accounted for more variance in the perceived stress‐mental well‐being relationship. SESRL reflects specific, actionable self‐regulatory skills, such as planning, monitoring, and strategy adjustment, that are particularly salient in the autonomous and cognitively demanding context of postgraduate study [56, 73, 96, 97]. Students with higher SESRL are more likely to engage in adaptive behaviors, such as help‐seeking [98], goal setting and organizing [73, 99], and emotional regulation [22], whereas students with lower SESRL may interpret challenges as indicative of inability and be more prone to disengagement [56]. SESRL may therefore serve as a key protective factor in postgraduate environments [17, 97, 100].

Collectively, these findings indicate that postgraduate well‐being is shaped by the interplay between stress appraisals and academic‐related efficacy beliefs. Perceived stress remains a key determinant of mental well‐being, though efficacy beliefs, particularly SESRL, appear to influence whether stress is internalized, amplified, or effectively managed. This reinforces the need for interventions that not only reduce harmful or excessive stress but also cultivate strong self‐efficacy beliefs, enabling postgraduate students to reframe expected and developmentally appropriate academic demands as challenges rather than threats.

### 4.4 Strengths and Limitations

This study contributes novel insights by focusing on UK postgraduate students and examining ASE and SESRL as mediators of the perceived stress‐mental well‐being relationship. Despite being conducted in a single institution, the sample was demographically diverse and broadly aligned with CU's postgraduate population. However, care should be taken when interpreting the findings, as this demographic profile may not necessarily reflect the broader UK postgraduate population. As with all cross‐sectional research, causal inferences cannot be drawn, and unmeasured psychological or contextual variables may also influence outcomes. Additionally, although adaptations were made to the ASE and SESRL scales for relevance to the university context, internal consistency remained strong and comparable to previous work [72, 73]. Finally, although we used the common heuristic of 15 participants per independent variable to provide an initial indication of sample size adequacy for regression models, we acknowledge that this rule is dated and that more rigorous, power‐analytic approaches are preferable. Nevertheless, given the medium‐to‐large associations observed among study variables and the use of 5000–sample bootstrapping to estimate indirect effects, the sample size (*N* = 334) was considered sufficient to produce stable and reliable mediation estimates in this study.

### 4.5 Implications

These findings highlight several opportunities for Higher Education institutions to enhance postgraduate well‐being by targeting both stress appraisal processes and the academic environments that shape them. Interventions that strengthen academic self‐efficacy, particularly SESRL, may help buffer the negative impact of perceived stress by increasing students; confidence in their capacity to manage expected academic demands. Universities are therefore encouraged to implement structured programmes that build core self‐regulatory competencies (e.g., planning, time management, adaptive study strategies, and motivation) while simultaneously supporting students' confidence in applying these skills [56].

In addition to skills‐focused provision, institutional practices should also prioritize supportive supervision and teaching approaches that reinforce mastery, capability, and academic progress. Supportive supervisory relationships, timely and constructive feedback, and teaching practices that normalize help‐seeking and promote adaptive coping may further enhance students' secondary appraisal processes [6, 34, 51, 59, 98]. Embedding efficacy‐building approaches within postgraduate education, rather than reliance on optional workshops alone, may help to promote sustained mental well‐being.

Finally, these findings highlight the value of universities addressing unnecessary or harmful academic stressors, alongside efforts to enhance students' perceived coping capacity and flexibility in responding to academic demands. Interventions that integrate skills development, supportive teaching and supervisory practices, and efficacy‐building may offer more lasting benefits for postgraduate well‐being.

## 5 Conclusion

This study demonstrates that perceived stress is strongly linked to poorer mental well‐being in UK postgraduate students, with both ASE and SESRL partially mediating this relationship. These findings highlight that postgraduate well‐being is influenced by both the demands students face and their confidence in managing those demands. For higher education institutions, this points to the need to prioritize support that builds students' self‐regulatory efficacy through structured training in adaptive study strategies alongside supervision and teaching practices that reinforce mastery and capability. By embedding interventions that strengthen both self‐regulation skills and the beliefs that these skills can be applied effectively, universities can better equip postgraduate students to cope with stress and promote more sustainable mental well‐being.

## Author Contributions

**Natalie Bisal:** writing – original draft, formal analysis, investigation, methodology, conceptualization, project administration, writing – review and editing. **Celine Brookes‐Smith:** conceptualization, methodology, investigation, project administration, writing – review and editing. **Riya Patel:** investigation, supervision, writing – review and editing. **Sally Sharp:** formal analysis, writing – review and editing. **Deborah Lycett:** conceptualization, methodology, formal analysis, funding acquisition, supervision, writing – review and editing. **Andy Turner:** conceptualization, methodology, funding acquisition, supervision, writing – review and editing. **Maxine Whelan:** conceptualization, methodology, supervision, writing – review and editing. All authors have read and approved the final version of the manuscript and agree to be accountable for all aspects of the work.

## Ethics Statement

Ethics approvals were obtained from Coventry University Research Ethics Committee (ref #P116128).

## Conflicts of Interest

The authors declare no conflicts of interest.

## Transparency Statement

The lead author Maxine Whelan affirms that this manuscript is an honest, accurate, and transparent account of the study being reported; that no important aspects of the study have been omitted; and that any discrepancies from the study as planned (and, if relevant, registered) have been explained.

## Data Availability

The authors have nothing to report.
